# Novel insights into chromosomal conformations in cancer

**DOI:** 10.1186/s12943-017-0741-5

**Published:** 2017-11-17

**Authors:** Ruobing Jia, Peiwei Chai, He Zhang, Xianqun Fan

**Affiliations:** 1 Department of Ophthalmology, Ninth People’s Hospital, Shanghai Jiao Tong University School of Medicine, Shanghai, People’s Republic of China; 2 Shanghai Key Laboratory of Orbital Diseases and Ocular Oncology, Shanghai, People’s Republic of China

**Keywords:** Chromosomal conformations, Cancer, Diagnosis, Treatment, Chromosome conformation capture

## Abstract

Exploring gene function is critical for understanding the complexity of life. DNA sequences and the three-dimensional organization of chromatin (chromosomal interactions) are considered enigmatic factors underlying gene function, and interactions between two distant fragments can regulate transactivation activity via mediator proteins. Thus, a series of chromosome conformation capture techniques have been developed, including chromosome conformation capture (3C), circular chromosome conformation capture (4C), chromosome conformation capture carbon copy (5C), and high-resolution chromosome conformation capture (Hi-C). The application of these techniques has expanded to various fields, but cancer remains one of the major topics. Interactions mediated by proteins or long noncoding RNAs (lncRNAs) are typically found using 4C-sequencing and chromatin interaction analysis by paired-end tag sequencing (ChIA-PET). Currently, Hi-C is used to identify chromatin loops between cancer risk-associated single-nucleotide polymorphisms (SNPs) found by genome-wide association studies (GWAS) and their target genes. Chromosomal conformations are responsible for altered gene regulation through several typical mechanisms and contribute to the biological behavior and malignancy of different tumors, particularly prostate cancer, breast cancer and hematologic neoplasms. Moreover, different subtypes may exhibit different 3D-chromosomal conformations. Thus, C-tech can be used to help diagnose cancer subtypes and alleviate cancer progression by destroying specific chromosomal conformations. Here, we review the fundamentals and improvements in chromosome conformation capture techniques and their clinical applications in cancer to provide insight for future research.

## Background

Gene expression in eukaryotic cells is regulated by many different complex mechanisms. In addition to epigenetic alterations (i.e., histone modifications, DNA methylation and noncoding RNA), chromosome conformation can influence gene function.

Chromosomes have unique, high-ordered structures that influence gene function. The higher-ordered genome structure often formed by hierarchical folding. First, the DNA double helix winds around an octamer of histone proteins to create the nucleosome. A beads-on-string fiber with a width of approximately 10 nm is then formed [1], followed by 30-nm fibers and global structures, and the length of the DNA chain folds to one-hundred-thousandth of the nonfolded size.

Proteins are involved in the formation of higher-ordered chromosome structures, such as chromosome loops. Some proteins, including special AT-rich sequence-binding protein-1 (SATB1), CCCTC-binding factor (CTCF) and cohesin, play key roles in disease development and recovery. Genes located at long distances from one another can interact through these known and unknown folding mechanisms to produce variable results.

### Techniques to detect chromosome conformation

To better study chromosome conformations, the following two types of techniques have been developed: observation methods and C-techs. Observation methods include fluorescence in situ hybridization (FISH), microscopy (light microscopy or electron microscopy), and nuclear ligation [2]; C-tech methods are more novel and technical. Chromosome conformation capture (3C) was invented in 2002 and was the first member of the C-tech family [3]. Subsequently, 3C has rapidly advanced to circular chromosome conformation capture (4C), chromosome conformation capture carbon copy (5C), high-resolution chromosome conformation capture (Hi-C), chromatin interaction analysis by paired-end tag sequencing (ChIA-PET), targeted chromatin capture (T2C), Capture-C and many others to meet a wider variety of needs (Table 1).

**Table 1:** Techniques used for chromosome conformation capture

Technology	3C	4C	5C	Hi-C	ChIA-PET
Data	One-one	One-all	Many-many	All-all	Many-many
Readout	PCR	Inverse PCR, microarrays, NGS	LMA, sequencing	Sequencing	Sequencing
Advantages	Simple, cheap	Relatively simple, goal-oriented	Explores interactions among many fragments, reduces bias, relatively targeted	Genome-wide interactions	Protein bonded interactions
Limitations	Laborious, only known combinations, poor resolution	Only chosen regions	Difficult to design hundreds of primers, analyzes only chosen fragments, some long-distance interactions are missing	Difficult to sequence and analyze, expensive	Analyzes only chosen protein
Ref.	[3, 10]	[12]	[17, 18, 142]	[19]	[24, 25]

Abbreviations: *PCR* Polymerase Chain Reaction, *NGS* Next-Generation Sequencing, *LMA* Ligation-mediated Amplification

Most of above mentioned technologies are based on 3C technology. After proteins are fixed and crosslinked with formaldehyde, chromatin is cut into 4- to 10-kb pieces [3] by 6-base recognizing enzymes, such as HindIII [3], EcoRI [4], BglII [5], DdeI [6], DpnII [7] and BamHI [8]. The use of a 4-base recognizing enzyme may be ideal for analyzing a narrow area, but these enzymes create too many small pieces. DNase I has been recently used to enhance the resolution [9]. One megabase is often considered the limit of 3C technologies, but larger distances [10] even those between chromosomes [11] have been reported. Semi-quantitative polymerase chain reaction (PCR) is always performed to verify the ligation junctions of interest. 3C technology is often used to analyze enhancer or insulator activities using CTCF but is limited in its ability to identify unknown regions.

4C technology enables the discovery of longer distance co-associations. Unlike the 6-base recognizing enzymes used in 3C, 4-base recognizing enzymes are used to cut chromosomes into 256-bp fragments. First, a single chromosome region of interest is selected, and global chromosomes associated with that region are sequenced. The target genes are amplified and sequenced, and global genome-wide interactions with the chosen fragment can be identified. This process is always described as one-to-all [12]. 4C technology also includes chromosome conformation capture on chromatin immunoprecipitation (ChIP) [13], which is similar to enhanced the chromosome conformation capture on ChIP (e4C) [14] approach that adds an optional ChIP step to test fragment binding to a chosen protein and can be more sensitive. Using 4C-seq [15], we can quickly and comprehensively identify the sequences of interest.

**Fig. 1:**
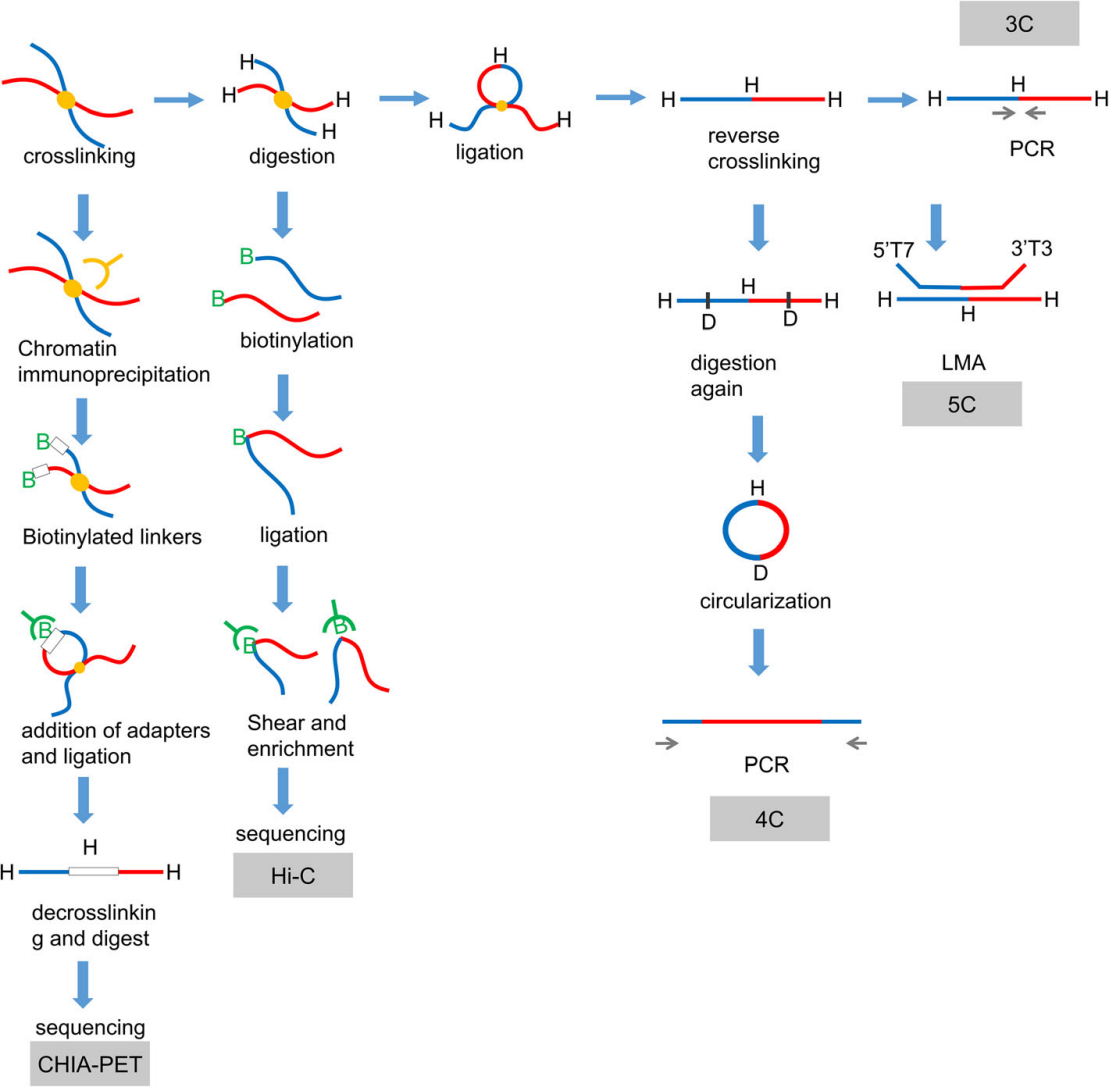
Overview of 3C-based C-techniques. After crosslinking, chromatin is digested into 4-10 kb pieces by restriction enzymes, followed by ligation and reverse crosslinking to change the crosses into lines. An additional digestion step is added in 4C. LMA is used instead of PCR, which is commonly used in 5C; biotinylation and streptavidin are used in Hi-C, and a chromatin immunoprecipitation (CHIP) step is added in ChIA-PET

5C can be used to map many interactions among several fragments in a region [16]. After the difficult step of designing hundreds of primers [17], ligation-mediated amplification (LMA) is commonly performed following 5C instead of PCR, which is commonly performed following 3C [18]. 5C can provide more detailed information and be used to construct a special network around genes of interest. Hi-C was designed in 2009 to recover ligated fragments using streptavidin to capture global genome-wide interactions, including long-distance interactions [19], and protocols and algorithms have demonstrated that it improves capture range and accuracy [20, 21]. Single-cell Hi-C can be used to analyze differences in chromosome conformation among cells and determine the uniqueness of cells [22]. In addition to chromosomes, ChIA-PET can be used to test global interactions with a chosen protein, such as CTCF [23] or polymerase II (Pol II) [24]. Moreover, new statistical patterns have been identified for ChIA-PET [25]. Additional new C-techs have been developed. Capture-C is a 3C-like technique with high resolution that can capture interactions in cis conformations using oligonucleotide probes [26]. T2C focuses on the target gene and explores its local interactions, thus reducing cost and labor [27], and is widely used to predict local loops [28]. Capture-Hi-C is a many-to-all Hi-C technique with higher resolution because it includes an additional step in which the sequences of interest (baits) are enriched [29]. RNA-guided chromatin conformation capture (R3C) is a new method that can be used to precisely study interactions between RNA and DNA [30, 31] (Fig. 1).

Other techniques, mainly developed prior to 3C techniques, can help to identify chromosomal interactions. FISH, microscopy (light microscopy or electron microscopy), and nuclear ligation [2] are widely used to observe and verify the higher-ordered chromosome organization. ChIA-PET is another advanced technique used to test global interactions with a chosen protein, such as CTCF [23] or DNA Pol II [24]. New statistical tools for ChIA-PET analysis have also been developed [25]. The 4D Nucleome Project provides a coherent view of how chromosome conformations change, providing insight beyond the static state [32]. One of the newest tools, genome architecture mapping (GAM), shows chromosomal contacts between long-range fragments by sequencing thin nuclear sections [33]. Currently, dCas9 capture with a biotinylated dCas9 step before 3C, RNA-seq or proteomics shows chromosomal interactions at a single-copy genomic locus and clarifies trans-regulatory factors [34].

### Characteristics and formation of chromosome loops

Because DNA is packaged and unpackaged in the nucleus, long-range intra- or inter-chromosomal interactions occur, and loops may be formed to stabilize these interactions. In fact, only 7% of loops occur between the two nearest genes. Of course, chromosome loops are not completely random. The specific DNA topology dictates the sequence [35], flexibility of the chromatin (the ring-closure probability) [36], and other dynamic factors, such as distance, energy [37] and negative DNA supercoiling [38]. A chromosome loop often contains at least two DNA sequences (for example, one sequence is an enhancer/silencer, and the other is a promoter) and some mediators. Although different cell types have different interactions, enhancers, CTCFs, and actively transcribed chromatin states rich in loops, 79% of loops span CTCF binding sites, which can prevent enhancer-promoter interactions without loops [39]. CTCF and cohesin are present in over 86% of loops. All loop domains are separated into 6 subcompartments by 8 histone markers, i.e., histone 3 lysine 36 trimethylation (H3K36me3), histone 3 lysine 27 trimethylation (H3K27me3), histone 3 lysine 4 methylation (H3K4me1), histone 3 lysine 4 dimethylation (H3K4me2), histone 3 lysine 4 trimethylation (H3K4me3), histone 3 lysine 9 trimethylation (H3K9me3), histone 3 lysine 79 dimethylation (H3K79me2), and histone 4 lysine 20 methylation (H4K20me1); the contact domains contain the same markers and change together [40]. In addition, these long-range interactions are inherited from sperm but not the ovum to regulate embryo development and growth [41, 42]. Loops are stable from the G1 to S and G2 phases of the cell cycle [43].

**Fig. 2:**
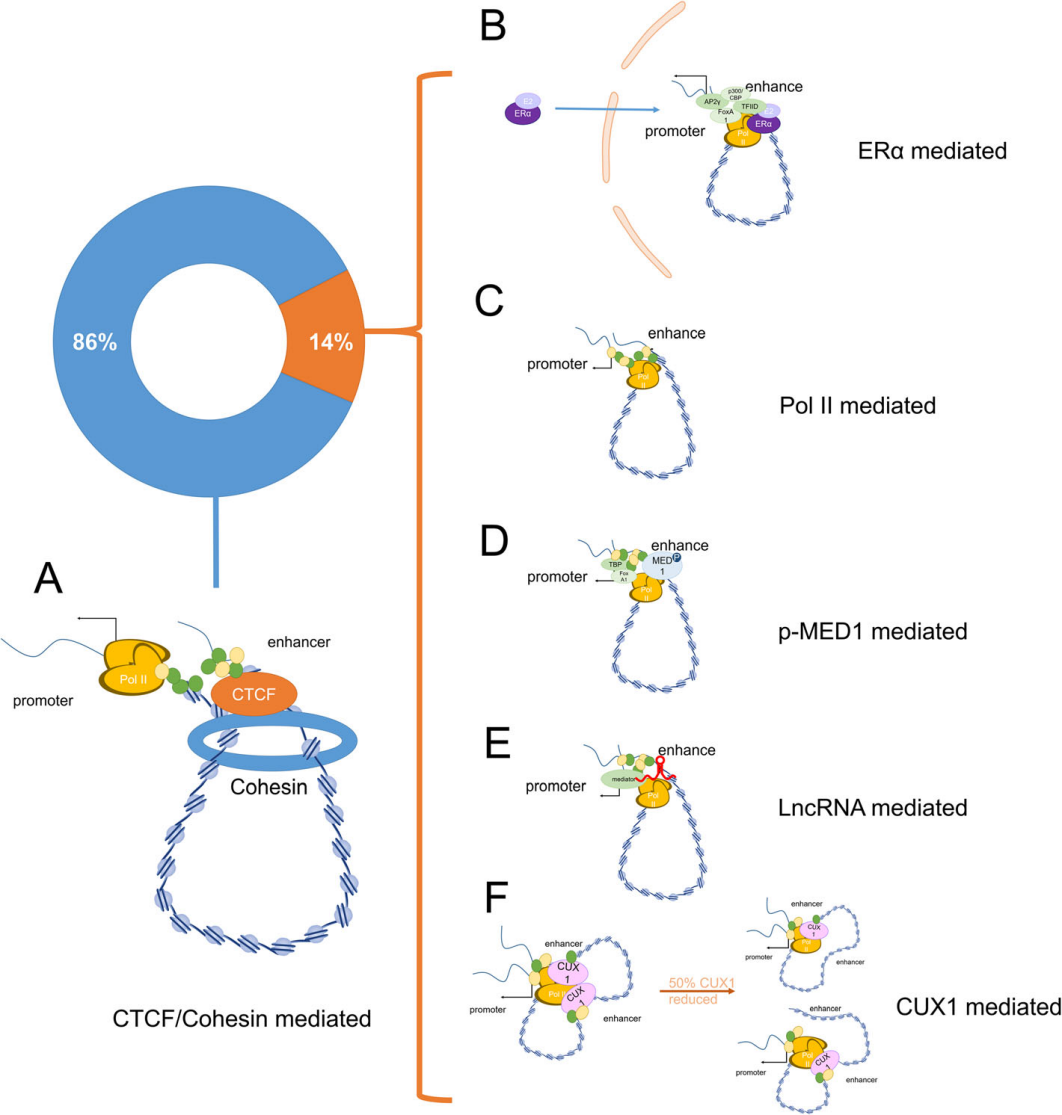
Several mechanisms for forming chromosomal loops in cancer. Pol II (orange), transcription factors (green) and co-transcription factors (yellow) are always recruited by long-range loop mediators in transcription factories. **a** Most loops are mediated by CTCF and cohesin, with or without other mediators. **b** ERα is another common mediator that functions after binding E2 and entering the nucleus. **c** Pol II can mediate long-range loops between promoters, suggesting that promoters sometimes act as enhancers. **d** MED1 acts as a chromosomal loop mediator only when it is phosphorylated. **e** Some lncRNAs can mediate chromosomal loops. **f** CUX1 is special in that it mediates the binding of two enhancers to one promoter, and when its expression is reduced to 50%, it mediates the binding of one enhancer to one promoter

The mechanism by which chromosomal loops alter gene regulation remains unclear, but several mechanisms have been proposed, particularly in cancer (Fig. 2). The examination of topologically associated domains (TADs) is another avenue for studying the relationship between chromosome loops and cancers. CTCF is enriched in the boundaries of TADs and can guide interactions by forming loops with cohesin between distantly located sequences [44] to influence gene expression [45]. These loops bind Pol II factories, including Pol II, transcription factors (TFs) and co-TFs [46]. CTCF and cohesin have been widely reported as the most common and important protein mediators [39, 40, 47]. Estrogen receptor α (ERα) is an estradiol (E2)-dependent mediator that binds E2 in the cytoplasm and is then translocated to the nucleus to activate Pol II and recruit forkhead box A1 (FoxA1) and activator protein (AP)-2γ [48–50]. Pol II can mediate interactions between enhancers and promoters and between distinct promoters [51], indicating that promoters can act as enhancers [24]. New mediators and mechanisms have been reported. Mediator complex subunit 1 (MED1) forms loops only when it is phosphorylated; phosphorylated MED1 facilitates loop formation by FoxA1, Pol II and TATA-box binding protein (TBP) [52]. Cut-like homeobox 1 (CUX1) is another mediator that can recruit Pol II via a new mechanism; at normal concentrations, CUX1 mediates interactions between promoters and several enhancers, but the number of binding sites decreases when the dosage of CUX1 is halved [53]. Binding of lncRNA to the polycomb repressive complex may increase the chance of interactions [54] or recruit a mediator protein to form long-range interactions [30]. Most loops form between promoters and promoters or promoters and enhancers, whereas a minority between promoters and insulators [55]; some loops are even involved in chromosomal translocations [56].

## Roles of chromosomal conformations in cancer

The rapid development of new techniques makes it possible to determine important roles of chromosomal conformations in many fields, especially in cancer. Specific proteins mediate the chromosome loops between different cancer-related genes (including their promoters) and an enhancer or a repressor to regulate gene transcription and influence the biological behaviors of carcinomas. In this review, we discuss the function of these loops and the possible clinical applications of these fatal chromosome conformations in diagnosis and treatment (Table 2).

**Table 2:** Summary of chromosomal interactions in cancer

Cancer	Typical interactions		Mediators	Therapeutic targets	Potential drugs	Ref
Prostate cancer	CRPC-specific enhancers	UBE2C	FOXA1 and phosphorylated MED1	PI3K/AKT/phosphorylated MED1 pathway	Carvacrol, ipatasertib, abiraterone acetate	[52]
	PSA enhancer	PSA promoter	AR/PCGEM1 and PRNCR1 (PCAT8)	AR	Sigma1 inhibitor, ASC-J9, cisplatin, niclosamide, EPI-001,	[62] [66]
	TFF3	ERG	ERG	ERG binding domain	ERG inhibitory peptides	[64]
	MOXD1	FYN/SERPINB9/HEY2				
	2p11.2	CAPG	/	/	/	[90]
	6q22.1	RFX6/GPRC6A				
	1q32.1	NFASC				
	8q24	MYC				
Breast cancer	IGFBP3	EGFR/MCF7/BCAS1-4	/	/	/	[68]
	GREB1	TFF1 promoter	ERα	/	/	[48–50]
	ERα	EGR1/TGFBR2/DAXX/BCL2L1	/	/	/	[74]
	ZEB2 promoter 1a	ZEB2 promoter 1b	AP-1	AP-1, ZEB2 and ERK/Akt	AP1-C301-S, SR 11302, valproic acid, U0126, miR-132	[77]
Hematologic neoplasms	GATA2 enhancer	EVI1	/	EVI1	Pyrrole-Imidazole Polyamide 1	[79]
	BCR	ABL	/	BCR-ABL fusion gene, BCL2	ZINC12891610 (hit2), venetoclax	[136]
	HoxA	Genes on chromosome 14	TFBS	/	/	[81]
	IGF1R promoter	IGF1R enhancer	IRAIN	/	/	[82]
	RUNX1 promoters	RUNX1 enhancer	RUNXOR	/	/	[56]
	Unknown promoters	Unknown enhancers	CUX1	CUX1	/	[53]
Colorectal cancer	MYC promoter	335-kb enhancer	CCAT1-L CTCF	/	/	[84]
	MYC promoter MECOM ETS1	rs6983267 (contains multiple enhancers) 3q26.2 11q23	CCAT1	/	/	[95]
Pancreatic cancer	rs386772267	DIS3 promoter	An allele-specific TF	/	/	[96]
Melanoma	MITF	BRN2/CDKN1A/TBX3	/	MITF	HIV1 protease inhibitor nelfinavir	[86]
Thyroid carcinoma	rs965513 mutant	FOXE1 and PTCSC2 promoter	/	/	/	[97]
Gliomas	FIPL1L1 enhancer	PDGFRA	CTCF altered by mutant IDH	/	/	[98]
	rs73001406	DDX6 promoter	/	/	/	[99]
Neurofibromatosis	NF1	1-Mb fragment nearby	/	/	/	[87]
Adenoid cystic carcinoma	MYB promoter	MYB enhancer	ACCMYB-TGFBR3 translocation	Super-enhancer BRD4	JQ1	[88]
Testicular germ cell tumors	Predicted GATA4 promoter 1q22 11q14.1	20q13.2 15q25.2 15q22.31	/	/	/	[100]

*PSA* prostate-specific antigen

### Chromosomal conformations in cancer

Two research strategies are used to identify new chromosomal conformations and their characteristics. C-techs are used to identify classical interactions that are mediated by typical proteins, and Hi-C is currently used to identify chromatin loops between cancer risk-associated single-nucleotide polymorphisms (SNPs) found by genome-wide association studies (GWAS) and their target genes.

#### Classical long-range interactions

ChIA-PET, CHIP-seq and 4C-seq are widely used to identify long-range interactions that are mediated by proteins or lncRNAs. In addition, most interactions are found in prostate cancer, breast cancer and leukemia, which will be discussed in detail in the following section.

The abnormal activation of the androgen receptor (AR) is a major cause of androgen-dependent prostate cancer (ADPC) and its advanced stage, i.e., castration-resistant prostate cancer (CRPC), in which the AR acts as a transcription factor. Some AR binding regions are far from the AR and associate with the AR through chromosome loops. In general, there are two ways for AR-driven chromosome looping to function in neoplasia [57]. First, AR can increase spatial association of the transmembrane protease, serine 2 (TMPRSS2) gene and ETS-related gene (ERG) and mediate TMPRSS2-ERG gene fusion [58], which is a clinical marker of prostate cancer. Moreover, the loops can associate long-range genes, which are often enhancers, with cancer-related gene promoters and change their expression [59]; this process is mediated by AR-collaborating TFs [60]. In addition, changes in certain active histone modifications, such as H3K4me1, H3K4me2 and histone H3 acetylation, are also important in forming chromosomal conformations. For example, ERG disrupts the AR by activating the H3K27 methyltransferase EZH2 [61]. Three CRPC-specific enhancers interact with the AR target gene ubiquitin-conjugating enzyme E2C (UBE2C) through chromosome loops mediated by FOXA1 and phosphorylated MED1, which leads to high expression of UBE2C and makes CRPC an incurable disease, unlike ADPC [52]. Similar loops can be observed between the AR binding sites and the promoters of prostate-specific antigen (PSA) [62], which is one of the most important genes in prostate cancer. Overexpression of ERG may also contribute to a change in chromosome topology [63]. In addition, the long-distance interactions among TFF3-ERG, MOXD1-FYN, MOXD1-SERPINB9, and MOXD1-HEY2 were specifically found in prostate cancer cells by a Hi-C analysis [64]. TADs are smaller and more plentiful in the prostate cancer cell genome, and the boundaries of these TADs attract more CTCF binding and H3K4me3 [65], providing more opportunities to form long-range interactions. In fact, these differences lead to cancer-related interactions between enhancers and promoters that occur in epigenetically deregulated domains [65]. lncRNAs are also involved; for example, PCGEM1 and PRNCR1 (PCAT8) mediate the enhancer-promoter chromosome loops and promote AR activation [66].

Chromosome conformations also influence tumorigenesis and breast cancer development. The long-range interactions of insulin-like growth factor-binding protein 3 (IGFBP3), a key gene in the development of breast cancer [67], are increased in breast cancer cells relative to those in normal cells [68]. The association between IGFBP3 with epidermal growth factor receptor (EGFR), another breast cancer-related gene, may contribute to tumorigenesis in a manner similar to the long-range interactions between IGFBP3 and breast carcinoma amplified sequence (BCAS1-4) in MCF7 cells [68]. ERα is another important mediator of long-range interactions in breast cancer. ERα was first reported to mediate long-distance interactions between GREB1 and the trefoil factor 1 (TFF1) gene promoter with the help of FOXA1 and AP-2γ [48–50]. Several other cofactors, including GATA3 [69], TLE1 [70], and PBX1 [71], also play roles in this dramatic process. The TF CTCF without cohesin binding sites is very rich in ERα binding sites and mediates enhancer-promoter loops [72]; a few sites have both cohesin and ERα but rarely CTCF [73]. A recent Hi-C experiment showed that many gene sites interacted with ERα, including cancer suppressor genes EGR1 and TGFBR2, apoptosis genes DAXX and BCL2L1, and densely mapped, distant estrogen-responsive elements located in 17q23 and 20q13 [74]. Another Hi-C study showed that runt-related transcription factor 1 (RUNX1) contributes to local interactions rather than long-range interactions in MCF7 cells [75]. Due to the development of ChIA-PET, more ERα-mediated interactions deeply involved in breast cancer have been identified [76].

Triple-negative breast cancer (TNBC) is a highly malignant breast cancer that is not associated with ERα. Activator protein 1 (AP1) is activated by tumor necrosis factor alpha (TNFα) and binds the long-range promoter of zinc finger E-box-binding homeobox 2 (ZEB2) to influence its expression, resulting in epithelial-mesenchymal transitions that are considered a potential novel therapeutic target for TNBC [77]. CTCF mediates the binding of the unmethylated imprinting control region to the IGF2 promoter region in the breast cancer MCF7 cell line [52]. An antisense noncoding RNA, i.e., IRAIN, is involved in the allele-switch pattern in breast cancer [31]. However, in many cases, more inter-chromosomal interactions appear in normal cells than in tumor cells [78].

The Philadelphia chromosome, which is an interchange between chromosomes 9 and 22 that produces a BCR-ABL fusion gene that can be found in almost all types of chronic myelogenous leukemia (CML), some types of acute lymphoblastic leukemia (ALL) and several types of acute myelocytic leukemia (AML), is a key example of the role of chromosome conformations in leukemia. Long-range interactions play an important role in this disease.

In AML with inv.(3)/t(3;3), an enhancer that interacts with GATA2 in normal cells, loses its connection to GATA2 and re-interacts with (active) EVI1, leading to cancer [79]. Translocation partner genes (TPGs) are located in transcriptional activity-related regions and mediate interactions among higher-order chromosome structures. This phenomenon was observed in both GM06990 and K562 cell lines [80]. In addition, it was determine the position of morbigenous translocations and revealed the global interactions of the HoxA gene on chromosome 7 [81]. lncRNAs, such as IRAIN, participate in the formation of intra-chromosomal loops; lncRNAs help the IGF1R promoter contact the enhancer, which is located 150 kb away [82]. RUNXOR can mediate the interaction between the RUNX1 promoters and its enhancer by forming a RUNX1 intra-chromosome loop and is involved in chromosomal translocation [56]. CUX1, a haploinsufficient TF, is a new mediator that contributes to long-range interactions between enhancers and sites close to the TSS in hematologic neoplasms and solid tumors; 32-49% of CUX1 co-localizes with RNA Pol II, and 34-70% of CUX1 co-localizes with EP300, a transcriptional co-activator. However, the cohesion complex appears more frequently, further demonstrating that CUX1 contributes to chromatin loops [53].

Chromosomal conformations play important roles in many malignant tumors. CTCF binding sites can change in colorectal cancer and are considered alterable chromosomal conformations [83]. In addition, lncRNA CCAT1-L together with CTCF can mediate a 335-kb interaction between the MYC promoter and its enhancer in colorectal cancer [84]. Microphthalmia-associated transcription factor (MITF) plays a major role in the development and metastasis of melanoma, another highly malignant tumor [85]. Three MITF-related chromosome loops (with BRN2, CDKN1A and TBX3) have been found in both cell lines and patient blood samples. This study was the first confirmation of long-range chromosome loops in melanoma and provided valuable prospects for diagnosis by examination of circulating immune cells [86]. A study investigating neurofibromatosis found a 1-Mb fragment containing the neurofibromatosis type I (NF1) gene that interacts with another nearby 1-Mb fragment recorded in the HindIII and NcoI-maps ([87]) using Hi-C data.

An interesting feedback loop exists in adenoid cystic carcinoma in which MYB protein-bound enhancers can interact long range to activate the promoter of MYB, and ACCMYB-TGFBR3 translocation also places the super-enhancer in contact with the promoter of MYB [88].

#### Loops involving cancer risk-associated SNPs

Cancer risk-associated SNPs are a leading area of study in oncomolecular biology, and long-range loops at these high-risk sites may be valuable. Some researchers combined Hi-C and occasionally 4C-seq with GWAS to identify additional novel and meaningful loops in which a new browser enables easy visual examination [89].

In prostate cancer, a capture Hi-C experiment revealed that some high-risk SNPs interact with their long-distance target genes, including CAPG, C2orf43, RFX6, NFASC, MYC and AGAP7P, through chromosome loops [90]. 4C-seq with GWAS was used to identify a risk locus for prostate cancers (LNCaP and C4-2B cells), and MYC and POU5F1B were ranked the highest, followed by CD96, PVT1, GSDMC, CXorf36, RRP12, USP14 and SMIN3, which may exhibit abnormal chromatin looping [91]. These techniques also revealed the following key pathways: the TFG-beta signaling pathway, p53 pathway and hypoxia response via HIF activation; which are considered highly important in the development of prostate cancer [91]. In breast cancer, Hi-C and GWAS have been combined to identify risk loci, including the protein-coding genes IGFBP5, KLF4, NSMCE2, and MYC, and the lncRNAs DIRC3, PVT1, and CCDC26, which are associated with CTCF [92, 93]. Capture Hi-C tech has also been applied to the study of colorectal cancer and used to identify long-range interactions between rs6983267 (later shown to contain multiple enhancers [94]) and a MYC promoter mediated by the MYC-related lncRNA CCAT1. Similar loops were identified between 3q26.2 and MECOM and between 11q23 and ETS1 [95]. The pancreatic cancer risk locus rs386772267 suppresses the expression of DIS3 via the loop between rs386772267 and the promoter of DIS3 with an allele-specific TF [96]. In papillary thyroid carcinoma (PTC), a combination of low-penetrance genes is critical, and one of these genes has SNP rs965513. Its mutants (i.e., SNPs rs7864322, rs12352658, rs7847449, and rs10759944) interact with a promoter shared by FOXE1 and PTCSC2 and act as enhancers [97]. Gliomas also display SNP-mediated interactions in which the oncogene PDGFRA is changed by CTCF-related chromosome folding [98]. Other interactions among enhancers, SNP rs73001406, and the DDX6 gene promoter affect cancer risk [99]. By combining GWAS and Hi-C, 19 new risk loci were identified in testicular germ cell tumors, including interactions between a predicted GATA4 promoter and 20q13.2, between 1q22 and 15q25.2, and between 11q14.1 and 15q22.31 [100].

### Chromosomal conformations lead to malignancy in cancer

Interactions between promoters and enhancers (or promoters sometimes) can influence gene expression. Some interactions have been reported to specifically change tumor behavior, and these effects depend on the target genes. Some target genes are known to be key in cancer: some are involved in chromosomal translocation [56], and others may be novel diagnostic targets.

Some examples include loops mediated by phosphorylated MED1 [52], the lncRNAs PCGEM1 and PRNCR1 [66], and MYB [88] that strongly promote cell growth. When the loops are disrupted, cancer growth is robustly inhibited in mouse models. The lncRNA IRAIN changes tumor migration ability [31]. When the interactions between GATA2 and EVI1 are disrupted, cancer cells have higher apoptosis rates [79], and CUX1 alters the expression of a cluster of genes and the cell cycle to promote cell proliferation [53]. However, long-range loops enable expression of PSA [62] and overexpression of oncogenic TFs [64].

## Potential diagnostic tools and therapeutic targets based on chromosomal conformations

Newly discovered loops could be used as novel diagnostic markers. These newly discovered interactions contribute to previously unknown pathways and may be potential therapeutic targets. There are many applications for the identification of chromosomal conformations in diagnosis and therapy.

### Diagnostic targets

Hi-C is a new tool that can be used to detect chromosomal aberrations and copy number variations in human cancer samples (for example, glioblastoma and anaplastic astrocytoma) to identify oncogene amplification, tumor suppressor gene deletion, fusion genes, and balanced/unbalanced structural rearrangements [101].

In prostate cancer, new loops with specific histone modifications may serve as new diagnostic markers; the TMPRSS2-ERG fusion has been used as a marker in diagnosis and risk assessment for many years, even before chromatin loops were identified as the culprit [102]. Some clinical trials have treated the TMPRSS2-ERG fusion gene as a biomarker; this gene can correct most of the false-negative results of the prostate cancer antigen 3 (PCA3) test, can act as a supplement to the serum PSA test [102, 103] and is associated with a poor outcome [104]. But deeper studies are needed to determine whether newly identified lncRNAs could be used as markers of breast cancer and AML.

In CML, the BCR-ABL fusion gene is often indicative of a poor prognosis, while other mutations lead to varying sensitivity to chemotherapy drugs according to European LeukemiaNet recommendations 2013 [105]. In addition, due to the characteristics of 3′ and 5′ TPG, disease-causing fusion genes may be predictive and used in diagnosis [80]. Human leukemia can be classified according to the different chromosome conformations. By mapping the HOXA gene cluster using the 5C technique and analyzing the gene cluster using 3D DNA disease-signature predictor, the MLL-fusion protein can be classified as wild-type, and the subtypes of leukemia can be classified according to their different MLL-fusion partners with high accuracy; thus, the MLL-fusion protein can be used as a novel marker, but this has been verified only in cell lines, and a clinical trial is necessary [106]. Studies have also identified new risk loci, such as NCK1, NCAPH2 and L3MBTL4, in B-cell malignancies (BCMs), including chronic lymphocytic leukemia, Hodgkin lymphoma and multiple myeloma [107]. Moreover, as CUX1 is inactivated in more than half of high-risk myeloid leukemia cases [108], it can be used as a biomarker of poor prognosis.

In melanoma, MITF-related loops identified in circulating tumor cells can be used to noninvasively detect cancer [86] and predict a poor prognosis [109], as shown in humans. In gliomas, 11q23.3 was identified as a susceptibility locus that is closely related to abnormal chromosome conformations [99], and rs965513 was identified as a risk factor in papillary thyroid carcinoma [97].

Many characteristic features have been found in different cancers and other diseases, such as autoimmune disease [110] and obesity [111]. An increasing number of candidate genes have been proposed, but they have not yet been commonly used as clinical markers. Applying these techniques remains challenging.

### Therapeutic targets

As previously discussed, many interactions and new functions of RNA and proteins have recently been found in many cancers, and these discoveries may present new therapeutic targets. In addition to small-molecule inhibitors of specific targets that may be predicted by drugs, plasmids carried by histidine-lysine peptide (HKP) enable the use of siRNA as a new agent for targeting specific sequences [112]. Here, we mention some therapeutic targets and drugs, and we describe completed clinical trials and studies with significant effects in vitro and in mice.

As androgen deprivation therapy for prostate cancer often leads to eventual resistance, there is an urgent need for new therapies. The chromosomal loops of UBE2C that are mediated by MED1 in both AR-positive and AR-negative CRPC, as discussed above, were reported to be a new therapeutic target in addition to the PI3K/AKT/phosphorylated MED1 pathway [52]. In clinical and experimental trials, many small-molecule inhibitors have been effective in targeting the AKT and P13K pathways in prostate cancer, including but not limited to carvacrol [113], ipatasertib [114], and abiraterone acetate [115]; phase III trials of abiraterone acetate have been completed and showed a benefit in CRPC patients pre- or post-chemotherapy [116]. Additionally, AR-targeted therapies have also had great success; the small molecule Sigma1 inhibitor suppresses AR function and further suppresses prostate cancer in vivo and in vitro [117]. Anti-AR drugs, such as cisplatin, niclosamide [118], ASC-J9 [119], EPI-001 [120], and even miRNA [121] or lncRNA [122, 123], also suppress prostate cancer [124]. Cisplatin was investigated in a multicenter phase II trial and was shown to be a safe, feasible and active therapy [125]. Gene fusions, such as TMPRSS2-ERG, are often the result of double-strand DNA breaks and error-prone DNA repair, mainly through the error-prone non-homologous end-joining pathway and we found that small-molecule inhibitors and siRNAs targeting this pathway decrease TMPRSS2-ERG gene fusion [126]. Other treatments with clinical potential include those that target the key enzyme EZH2 via its inhibitors DZNep [127] and GSK126 [128]; similarly, ERG inhibitory peptides (EIPs) specifically target the ERG binding domain [129].

In TNBC, C-techs have not only provided evidence that AP1 mediates the loop of the ZEB2 promoters but also identified AP-1, ZEB2, and ERK/Akt signaling as potential drug targets [77]. Therefore, the AP1 inhibitors AP1-C301-S and SR 11302 [130]; ERK/Akt inhibiting drugs, such as valproic acid and U0126 [131]; and miR-132, which targets ZEB2 [132], may be potential drugs; a phase I/II trial of valproic acid was completed and showed the ability of valproic acid to potentiate the effects of epirubicin [133].

Inhibitors that target key proteins in chromosome loops may be novel drugs for the treatment of hematologic neoplasms. For example, pyrrole-imidazole polyamide 1 can target and decrease the expression of EVI1 [134], while GZD856 [135], ZINC08764498 and ZINC12891610 are unique Bcr–Abl fusion gene inhibitors [136]. Venetoclax inhibits BCL2 (an overexpressed cancer-related gene in BCM) [107], and it has received global approval as an oral medicine while under study in two phase III trials [137].

In melanoma, drugs targeting MITF, such as the HIV1 protease inhibitor nelfinavir, can decrease the formation of loops [138]. BET bromodomain inhibitors, such as JQ1 [139], which can suppress the super-enhancer BRD4 [140], may be developed into treatments for ACC. Moreover, the cancer inhibiting role of DIS3 could be critical for the design of new drugs [96].

However, even with such promising targets and treatments, much work is needed to identify drugs that target each loop of interest.

## Conclusions

Long-range interactions play an increasingly significant role in the development of malignant tumors. An increasing number of long-range interactions have been discovered in prostate cancer, breast cancer, hematologic neoplasms and other cancers and may serve as new diagnostic and therapeutic targets.

Currently, we can study interactions between only two fragments of DNA due to the limitations of available techniques. To determine the underlying mechanisms of cancer onset and progression, we must develop tools for broader analysis. The question of how chromosomes fold and loop is fascinating and has attracted the attention of scientists from many fields, including those studying cancer. As discussed above, we know that risk loci and certain key genes are “hot spots” in which chromosome conformation changes can be reliably found. In addition to these sites, many different mutations of oncogenes and tumor suppressor genes have been detected in cancers. We can surmise that typical cancer-related genes may encompass a new category of chromosome-conformation-related protein binding sites; the binding of these sites leads to chromosome conformation changes, subsequent gene function changes and possibly new pathogenic mechanisms. The use of C-techs may also help to identify more new cancer-related genes. We can then concentrate on the development of small molecule drugs that target these new genes or mechanisms because novel targeting drugs are at the forefront of cancer research and greatly contribute to medical development.

It is likely that there are more unknown proteins and RNAs (or known proteins and RNAs with unknown functions) that mediate these interactions; we call these proteins and RNAs new loop organizers. These undiscovered loop organizers might underlie more new mechanisms and pathways. New loop organizers and their foundations alike may yield new diagnostic and therapeutic targets. Moreover, there are many elements involved in forming long-range interactions, including lncRNAs and proteins, that cannot all be targeted simultaneously in new therapies. However, key elements that alter other elements when targeted must be identified to increase the efficacy of oncotherapy, similar to the idea of “one vehicle with multiple satellites”. There have been some attempts to target key lncRNAs in oncotherapy [141], and if we develop new therapies related to high-order chromatin conformations based on this idea, we may create a closer link between chromatin conformation and clinical applications.

Future studies should explore how chromosome conformation changes can become a tool for clinical diagnosis and improved therapies.

## Abbreviations

3C: Chromosome conformation capture
4C: Circular chromosome conformation capture
5C: Chromosome conformation capture carbon copy
ADPC: Androgen-dependent prostate cancer
ALL: Acute lymphoblastic leukemia
AML: Acute myelocytic leukemia
AR: Androgen receptor
BCAS1-4: Breast carcinoma amplified sequence
BCM: B-cell malignancies
ChIA-PET: Chromatin interaction analysis by paired-end tag sequencing
CHIP: Chromatin immunoprecipitation
CML: Chronic myelogenous leukemia
CRPC: Castration-resistant prostate cancer
CTCF: CCCTC-binding factor
CUX1: Cut-like homeobox 1
E2: Estradiol
e4C: Enhanced chromosome conformation capture-on-ChIP
EGFR: Epidermal growth factor receptor
ERG: ETS-related gene
ERα: Estrogen receptor α
FISH: Fluorescence in situ hybridization
FoxA1: Forkhead box A1
GAM: Genome architecture mapping
GATA1: GATA-binding factor 1
GWAS: Genome-wide association studies
H3K27me3: Histone 3 lysine 27 trimethylation
H3K36me3: Histone 3 lysine 36 trimethylation
H3K4me1: Histone 3 lysine 4 methylation
H3K4me2: Histone 3 lysine 4 dimethylation
H3K4me3: Histone 3 lysine 4 trimethylation
H3K79me2: Histone 3 lysine 79 dimethylation
H3K9me3: Histone 3 lysine 9 trimethylation
H4K20me1: Histone 4 lysine 20 methylation
Hi-C: High-resolution chromosome conformation capture
HKP: Histidine-lysine peptide
IGF2: Insulin-like growth factor 2
IGFBP3: Insulin-like growth factor-binding protein 3
LMA: Ligation-mediated amplification
lncRNAs: Long noncoding RNA
MED1: Mediator complex subunit 1
MITF: Microphthalmia-associated transcription factor
NGS: Next-generation sequencing
PCR: Polymerase chain reaction
Pol II: Polymerase II
PSA: Prostate-specific antigen
PTC: Papillary thyroid carcinoma
R3C: RNA-guided chromatin conformation capture
RUNX1: Runt-related transcription factor 1
SATB1: Special AT-rich sequence-binding protein-1
SNPs: Single-nucleotide polymorphisms
T2C: Targeted chromatin capture
TAD: Topologically associated domain
TBP: TATA-box binding protein
TF: Transcription factor
TFF1: Trefoil factor 1
TNBC: Triple-negative breast cancer
TNFα: Tumor necrosis factor alpha
UBE2C: Ubiquitin-conjugating enzyme E2 C
ZEB2: Zinc finger E-box-binding homeobox 2
